# Yuzu and Hesperidin Ameliorate Blood-Brain Barrier Disruption during Hypoxia via Antioxidant Activity

**DOI:** 10.3390/antiox9090843

**Published:** 2020-09-09

**Authors:** Bo Kyung Lee, Soo-Wang Hyun, Yi-Sook Jung

**Affiliations:** 1 College of Pharmacy, Ajou University, Suwon 16499, Korea, dix07@naver.com (S.-W.H.); 2 Research Institute of Pharmaceutical Sciences and Technology, Ajou University, Suwon 16499, Korea

**Keywords:** blood–brain barrier (BBB), reactive oxygen species (ROS), hypoxia, yuzu, hesperidin (HSP), tight junction (TJ)

## Abstract

Yuzu and its main component, hesperidin (HSP), have several health benefits owing to their anti-inflammatory and antioxidant properties. We examined the effects of yuzu and HSP on blood–brain barrier (BBB) dysfunction during ischemia/hypoxia in an in vivo animal model and an in vitro BBB endothelial cell model, and also investigated the underlying mechanisms. In an in vitro BBB endothelial cell model, BBB permeability was determined by measurement of Evans blue extravasation in vivo and in vitro. The expression of tight junction proteins, such as claudin-5 and zonula occludens-1 (ZO-1), was detected by immunochemistry and western blotting, and the reactive oxygen species (ROS) level was measured by 2′7′-dichlorofluorescein diacetate intensity. Yuzu and HSP significantly ameliorated the increase in BBB permeability and the disruption of claudin-5 and ZO-1 in both in vivo and in vitro models. In bEnd.3 cells, yuzu and HSP were shown to inhibit the disruption of claudin-5 and ZO-1 during hypoxia, and the protective effects of yuzu and HSP on claudin-5 degradation seemed to be mediated by Forkhead box O 3a (FoxO3a) and matrix metalloproteinase (MMP)-3/9. In addition, well-known antioxidants, trolox and N-acetyl cysteine, significantly attenuated the BBB permeability increase, disruption of claudin-5 and ZO-1, and FoxO3a activation during hypoxia, suggesting that ROS are important mediators of BBB dysfunction during hypoxia. Collectively, these results indicate that yuzu and HSP protect the BBB against dysfunction via maintaining integrity of claudin-5 and ZO-1, and these effects of yuzu and HSP appear to be a facet of their antioxidant properties. Our findings may contribute to therapeutic strategies for BBB-associated neurodegenerative diseases.

## 1. Introduction

The blood–brain barrier (BBB) is a functional and structural barrier in the cerebrovascular endothelium that maintains brain homeostasis by regulating the influx of many compounds and pathogens from blood circulation into brain tissue [1]. A normal BBB is characterized by endothelial cells with a few pinocytic vesicles, abundant mitochondria, and interendothelial tight junction (TJ) proteins. TJ proteins, such as zonula occludens-1 (ZO-1) and claudin-5 in the cerebrovascular endothelium, form the initial BBB between the systemic circulation and the central nervous system (CNS) [2]. Under normal conditions, the BBB acts as a gatekeeper for the CNS, effectively protecting the brain from detrimental substances [3]. However, the BBB is known to be disrupted under pathological conditions, such as neurodegenerative diseases. Loss of BBB integrity not only favors the formation of vasogenic cerebral damage but also promotes the infiltration of circulating leukocytes, which exaggerate BBB breakdown and cerebral inflammation [4]. Recently, BBB breakdown has been reported to be a risk factor for vascular cognitive impairment (VCI), leading to vascular dementia [5,6,7]. Moreover, BBB dysfunction has also been recognized to play a critical role in the pathogenesis of Alzheimer’s disease (AD) [8,9,10]. AD patients demonstrate morphological abnormalities of the neurovascular unit, such as endothelial atrophy, degeneration of smooth muscle cells, and disruption and thickening of the basement membrane [11].

There have been many studies showing that reactive oxygen species (ROS) contribute to BBB breakdown and subsequent inflammation in the brain [12]. It has been demonstrated that neuroinflammation caused by hypoxia stimulates microglia/macrophages to release ROS and proteases, such as matrix metalloproteinases (MMPs), which subsequently increase BBB permeability and attack myelinated fibers, finally resulting in vascular dysfunction in the brain. It is also known that large amounts of ROS directly contribute to the loss of endothelial cell interactions and affect BBB integrity [4]. Several studies have also shown that increased BBB permeability caused by oxidative stress [13,14] is generally related to alterations in claudin-5 and ZO-1, however, the mechanisms underlying these processes have not been fully elucidated.

Yuzu (*Citrus junos* Sieb. ex Tanaka), a citrus plant native to northeast Asia, is commonly used as traditional medicine [15]. Yuzu contains higher amounts of vitamin C and phenolics than other citrus fruits, offering better health benefits owing to its anti-inflammatory and antioxidant properties. Hesperidin (HSP), one of the main components of yuzu, is a member of the flavonoid group of polyphenols [16]. Our previous study demonstrated that yuzu and HSP have an anti-thrombotic effect [15] and a cardioprotective effect in rats [17,18,19]. Several studies have reported that the health benefits of yuzu are largely attributed to the presence of relatively high concentrations of antioxidant compounds such as HSP [20,21]. However, little information is available about the effects of yuzu and HSP on BBB dysfunction during hypoxia/ischemia. In the present study, we examined the effects of yuzu and HSP on hypoxia-induced BBB hyperpermeability and TJ disruption, and whether the underlying mechanisms for the protective effects of yuzu and HSP include their antioxidant activity.

## 2. Materials and Methods

### 2.1. Reagents 

Ethanolic extracts of yuzu and its HPLC analysis were provided by Prof. SW Park at Konkuk University (Seoul, Korea) (Figure S1 and Table S1). Briefly, yuzu was extracted with ethanol and lyophilized to remove solvent (Supplementary Methods). Yuzu extract was dissolved in saline (0.9% NaCl) for the in vivo study. HSP isolated from citrus species was purchased from Sigma-Aldrich (St. Louis, MO, USA) (Figure S2). Trolox and N-acetyl cysteine (NAC) were purchased from Tocris Bioscience (Ellisville, MO, USA). The following commercial antibodies were used: claudin-5 and ZO-1 (Invitrogen, Carlsbad, CA, USA), β-actin and Foxo3a (Cell Signaling Technology, Inc., Danvers, MA, USA), and UEA-1 (Sigma-Aldrich, St. Louis, MO, USA). All other chemical reagents were purchased from Sigma-Aldrich and were of analytical or HPLC grade.

### 2.2. Transient Brain Ischemia Model

All animal protocols were approved by the Institutional Animal Care and Use Committee of the Ajou University Medical School (No. 2016-0007). All experiments were performed on Institute of Cancer Research (ICR) mice (male, 8 weeks old, 30–35 g; Orient Bio, Seongnam, Korea), housed under controlled temperature (23 ± 1 °C) and humidity (50 ± 5%). Five to seven mice were used for each experimental group. The ICR mice were anesthetized with a 1.0 mL i.p. injection of an anesthetic cocktail consisting of ketamine (100 mg/kg, Yuhan Corp., Seoul, Korea) and xylazine (10 mg/kg, Bayer Korea Ltd., Seoul, Korea) before surgery. Brain ischemia was induced by occluding the left middle cerebral artery (MCAO) using an intraluminal filament technique described previously [22]. The thread was carefully withdrawn 2 h after MCAO.

### 2.3. Measurement of Brain Water Content

Brain water content was measured using a procedure described previously [22]. Briefly, after a 24 h reperfusion, the brain was removed and separated into left and right hemispheres. The whole left hemisphere was weighed, dried for 48 h at 60 °C, and weighed again to obtain the dry weight. Brain water content was calculated as: (wet weight-dry weight)/wet weight. All mice were administered i.v. yuzu or HSP in 50% polyethylene glycol (PEG; 1, 3, or 10 mg/kg) before MCAO.

### 2.4. Measurement of Evans Blue Extravasation 

Evans blue extravasation was measured using a procedure described previously [22]. Briefly, after 10 min of reperfusion, mice were given i.v. injections of Evans blue dye (4 mL/kg 2% Evans blue in saline: Sigma-Aldrich, St. Louis, MO, USA). After 24 h of reperfusion, the brain was removed from transcardially perfused mice with saline and separated into left and right hemispheres. The whole left hemisphere was weighed, placed in 400 μL pure formamide (Sigma Aldrich, St. Louis, MO, USA), and incubated for 72 h at 50 °C. The optical density of the formamide solution was measured at 620 nm. All mice were treated with i.v. Yuzu or HSP in 50% PEG (10 mg/kg) before MCAO.

### 2.5. Immunohistochemistry

Immunostaining for claudin-5 and ZO-1 was performed using a procedure described previously [22]. Briefly, brains were removed from mice who underwent transcardial saline perfusion after 24 h of reperfusion. The brain tissues were fixed in 4% paraformaldehyde (PFA; Sigma-Aldrich, St. Louis, MO, USA) for 24 h at 4 °C and then transferred to 30% sucrose in phosphate buffered saline (PBS) for 48 h at 4 °C. The tissues were embedded in Tissue-Tek optimal cutting temperature (OCT) compound (Sakura Finetek Inc, Torrance, CA, USA), frozen at −70 °C for 30 min, and sectioned at 20 μm using a frozen section machine (Meyer Instruments, Houston, TX, USA) at −33 °C; the sections were mounted on poly-D-lysine-coated glass slides and soaked in 3% bovine serum albumin (BSA) blocking solution at 25 °C. Sections were incubated with anti-ZO-1 antibody (1:50, Invitrogen, Carlsbad, CA, USA) or anti-claudin-5 antibody (1:100, Invitrogen) overnight and then with secondary antibody labeled with Alexa 568 (1:500; Invitrogen). Finally, the sections were incubated with fluorescein isothiocyanate (FITC)-labeled *Ulex europaeus* agglutinin-1 (UEA-1; Sigma Aldrich). All samples were observed under a Nikon C2 confocal microscope (Nikon, Tokyo, Japan).

### 2.6. bEnd.3 Cell Culture

The bEnd.3 cells were purchased from the American Type Culture Collection (ATCC; Manassas, VA, USA) and grown in Dulbecco’s modified Eagle’s medium (DMEM; Gibco-BRL, Grand Island, NY, USA) with 10% fetal bovine serum (FBS). Confluent bEnd.3 cells were incubated in an anaerobic chamber (Forma Scientific, Marietta, OH, USA) at 37 °C in an atmosphere of 5% CO_2_, 10% H_2_, and 85% N_2_ in glucose-free DMEM that had been saturated with N_2_ gas for 30 min as a hypoxia challenge.

### 2.7. Permeability Assay In Vitro

The bEnd.3 cells were grown on the inside of gelatin-coated Transwell inserts (0.4 µm, Corning-Costar, Corning, NY, USA) and exposed to hypoxic conditions for 4 h. Next, 165 μg/mL Evans blue-0.1% BSA (Sigma Aldrich) or sodium fluoride (NaF; Sigma Aldrich) were added to the upper chamber 1 h before measurements. The intensities of the diffused Evans blue-0.1% BSA or NaF in the lower chamber were measured at 650 nm and 485/535 nm, respectively. Results are expressed as the ratio of Evans blue-0.1% BSA or NaF concentration in the lower chamber to the total concentration of Evans blue-0.1% BSA or NaF added to the upper chamber at the beginning of the experiment. Trans-endothelial electrical resistance (TEER) across the membrane was measured using a Millicell ERS-2 Voltohmmeter (Millipore Co., Billerica, MA, USA). The gelatin-coated Transwell inserts were placed in 24-well plates containing culture medium and used to measure background resistance. The resistance measurements of these blank filters were subtracted from those of filters with cells. Values were measured as Ωcm^2^, based on the culture inserts. All cells were pretreated with HSP (3, 10, or 30 μg/mL) 30 min before hypoxia exposure.

### 2.8. Immunocytochemistry 

Immunocytochemistry was performed by modifying procedures that have been previously described [23]. The samples were fixed with methanol at 25 °C and then soaked in 3% BSA blocking solution, also at 25 °C. The samples were incubated with anti-ZO-1 antibody (1:400, Invitrogen), anti-claudin-5 antibody (1:400, Invitrogen), or anti-FoxO3a antibody (1:400, Cell Signaling Technology, Inc., Danvers, MA, USA) overnight and then with secondary antibody labeled with Alexa 488 (1:500, Invitrogen). Finally, the samples were incubated with Hoechst 33258. All samples were observed under a Nikon C2 confocal microscope and all cells were pretreated with 30 μg/mL HSP 30 min before hypoxia exposure.

### 2.9. Western Blot Analysis

Western blot analysis was performed by modifying a procedure described previously [24]. The cells were lysed and centrifuged at 14,000 rpm for 15 min and the supernatant was collected to obtain the whole-cell lysate. The proteins were separated by sodium dodecyl sulfate-polyacrylamide gel electrophoresis (SDS-PAGE) and reacted with anti-ZO-1 antibody (1:1000, Invitrogen) or anti-claudin-5 antibody (1:1000, Invitrogen) overnight. All samples were analyzed using the LAS 4000 mini (Fuji Photo Film, Tokyo, Japan). All cells were pretreated with 30 μg/mL HSP 30 min before hypoxia exposure. 

### 2.10. Subcellular Preparation for Membrane and Cytoskeleton Fraction

After hypoxia exposure, the cells were lysed using different lysis buffers, and lysates were extracted using different procedures. Briefly, the cells were incubated in lysis buffer A (20 mM Tris-HCl, pH 7.4, 250 mM sucrose, 1 mM EDTA, 0.1 mM NaF, 0.2 mM Na_3_VO_4_, 0.5 mM phenylmethylsulfonyl fluoride, 0.01 mM leupeptin, and 0.01 mg/mL aprotinin) for 30 min on ice, and lysates were centrifuged at 200,000 × g in a Beckman Optima TL Ultracentrifuge (Beckman Coulter, Brea, CA, USA) at 4 °C for 10 min. The supernatants (cytosol fractions, CFs) were removed and the remaining pellets were resuspended with lysis buffer B (lysis buffer A containing 1% Triton X-100) and incubated on ice for 1 h. The suspensions were then centrifuged as mentioned above, and the supernatants (membrane fractions, MFs) were removed. The pellets were resuspended in lysis buffer C (lysis buffer B containing 1% sodium dodecyl sulfate (SDS)) and incubated on ice for 1 h. The suspensions were centrifuged as above, and the supernatants (actin cytoskeleton fractions, ACFs) were removed. Samples of each ACF were diluted with SDS sample buffer and incubated at 100 °C for 10 min.

### 2.11. Reverse Transcription Polymerase Chain Reaction 

Reverse transcription polymerase chain reaction (RT-PCR) was performed by modifying procedures described previously [25]. Briefly, total RNA was isolated for reverse transcription (RT). After RT, cDNA was amplified using specific primers for MMP-3 (NM_010809, forward, 5′-TTG CCA ACC TGC GTA TCT GT-3′; reverse, 5′-TCC CAA GGA TGC CTA GCT CT-3′) and MMP-9 (NM_013599, forward, 5′-CAG CCA GAC ACT AAA GGC CA-3′; reverse, 5′-CCT CGA AGG TGA AGG GAA AG-3′). All samples were analyzed using GelDoc^TM^ (Bio-Rad, Hercules, CA, USA).

### 2.12. siRNA Transfection

A procedure described previously was modified and used to transfect siFoxO3a [26]. Briefly, bEnd.3 cells were transiently transfected in siRNA transfection medium with siFoxO3a using an siRNA transfection reagent (Santa Cruz Biotechnology, Inc., Dallas, TX, USA). 

### 2.13. 2,2-Diphenyl-1-Picrylhydrazyl Assay

The free radical scavenging activity of the yuzu and HSP was determined in vitro using a 2,2-diphenyl-1-picrylhydrazyl (DPPH; Sigma Aldrich, St. Louis, MO, USA) assay as per a method described preciously. The protocol for the DPPH radical scavenging activity test was adapted from Brand-Williams et al. (1995), with some changes [27]. Aliquots of 100 μL of a methanolic solution containing different concentrations ranging from 0.3–30 mg/mL of yuzu and 1 to 100 μg/mL of HSP were added to 100 μL of 250 μM methanolic solution of DPPH. Absorbance at 517 nm was determined after 30 min, and the percent inhibition activity was calculated. The percentage (%) of scavenging of the DPPH-free radical was calculated using the formula: (A0−A1)/A0 × 100, where A0 is the absorbance of the control and A1 is the absorbance of the extract/standard. 

### 2.14. 2’7’-Dichlorofluorescein Diacetate Fluorescence Assay

Changes in ROS levels were measured using 2′7′-dichlorofluorescein diacetate (DCF-DA) dye (10 μM) (Molecular Probes, Eugene, OR, USA), a ROS-selective fluorescent indicator. Briefly, bEnd.3 cells were rinsed with 4-(2-hydroxyethyl)-1-piperazineethanesulfonic acid (HEPES)-controlled salt solution (HCSS) (120 mM NaCl, 5 mM KCl, 1.6 mM MgCl_2_, 2.3 mM CaCl_2_, 15 mM glucose, 20 mM HEPES, and 10 mM NaOH, pH 7.4) and loaded with DCF-DA dye (10 μM) and Pluronic F-127 (10 μM) for 30 min at 37 °C in HCSS before hypoxia exposure. After the hypoxia treatment, intracellular ROS release induced by 1% Triton-X100 was measured at an excitation wavelength of 485 nm and an emission wavelength of 530 nm.

### 2.15. Statistical Analysis 

All data are expressed as mean ± standard error of the mean (SEM). Comparisons were conducted using Student’s *t*-tests and/or one-way analysis of variance (ANOVA). A value of *p* < 0.05 was considered significant. 

## 3. Results

### 3.1. Effects of Yuzu and HSP on Brain Edema and BBB Dysfunction in a Mouse MCAO Model

**Figure 1:**
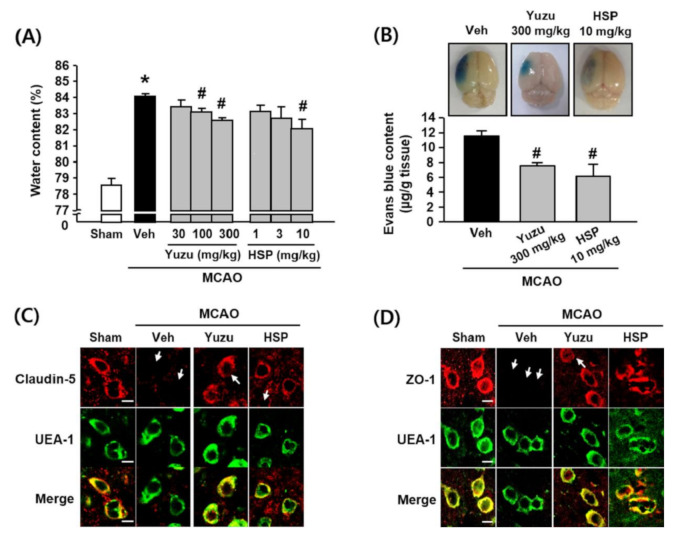
Effects of yuzu and HSP on water content and BBB (Blood-Brain Barrier) dysfunction in a mouse MCAO model. (**A**) Quantitative analysis of water content in brain tissues. Water content was measured 24 h after MCAO in ischemic hemispheres. All mice were intravenously treated with yuzu (30, 100 or 300 mg/kg) or HSP (1, 3, or 10 mg/kg) 30 min before MCAO. Data are shown as mean ± SEM. (*n* = 5–7). (**B**) Top, representative photographs of the whole brain. Evans blue extravasation was observed in the ischemic hemispheres as blue areas in the whole left brain. Bottom, quantitative analysis of Evans blue content in brain tissues. Data are shown as mean ± SEM (*n* ≥ 5). * *p* < 0.05 vs. sham, # *p* < 0.05 vs. Veh. (**C**,**D**) Representative fluorescence images of (**C**) ZO-1 and (**D**) claudin-5. Immunostaining for ZO-1 and claudin-5 were performed in peri-ischemic regions of ischemic hemispheres at 24 h of reperfusion. All mice were administered i.v. yuzu (300 mg/kg) or HSP (10 mg/kg) 30 min before MCAO. The endothelium was identified using the endothelial marker, UEA-1. ZO-1 and claudin-5 were located in brain endothelium, as shown in the merged figures. Arrows indicate regions of TJ disruption. The images are representative of four separate experiments. Scale bar, 20 µm. MCAO: middle cerebral artery occlusion; Veh: vehicle; HSP: hesperidin.

As shown in Figure 1A, the water content was greater in the ischemic hemispheres (84.13 ± 0.17%) of the MCAO group than in the sham group (78.61 ± 0.41%), and this increase was significantly attenuated by administering 100 and 300 mg/kg of yuzu (i.p., 83.14 ± 0.23% and 82.62 ± 0.16%, respectively) or 10 mg/kg HSP (i.p., 82.12 ± 0.54%). To investigate the effects of yuzu and HSP on BBB dysfunction in an MCAO animal model, we examined cerebral microvasculature permeability using the Evans blue extravasation assay [28]. As shown in Figure 1B, Evans blue extravasation was greater in the ischemic group (11.58 ± 0.66 μg/g tissue) than in the sham group (2.26 ± 0.35 μg/g tissue). This increase was significantly attenuated by 300 mg/kg yuzu and 10 mg/kg HSP (7.56 ± 0.40 μg/g tissue and 6.18 ± 1.61 μg/g tissue, respectively).

TJs comprise transmembrane proteins between BBB endothelial cells and play a critical role in regulating BBB paracellular permeability [29]. We performed immunohistochemistry to assess the effects of yuzu and HSP on the disruption of TJs (claudin-5 and ZO-1) in a mouse MCAO model. Our results showed that the immunostaining of claudin-5 and ZO-1 were normal in sham-operated brain tissues, whereas abnormally discontinuous staining for claudin-5 and ZO-1 was observed around the vessels of ischemic hemispheres. The disruption of claudin-5 and ZO-1 was prevented by yuzu (300 mg/kg) and HSP (10 mg/kg) in the peri-ischemic region of the MCAO group (Figure 1C,D).

### 3.2. Effects of Yuzu and HSP on BBB Permeability during Hypoxia in bEnd.3 Cells

**Figure 2:**
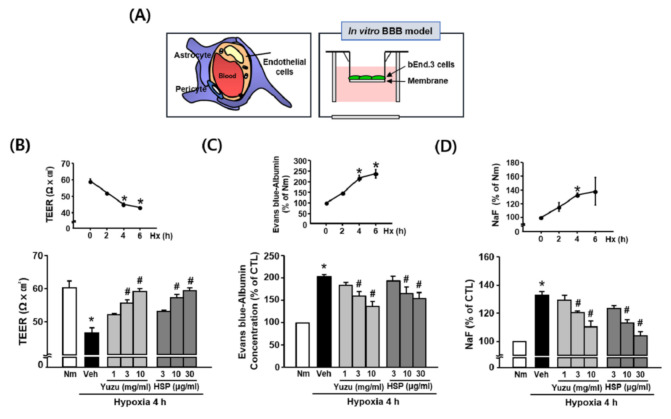
Effects of yuzu and HSP on BBB permeability during hypoxia in bEnd.3 cells. (**A**) Schematic representation of in vitro model of BBB. (**B**–**D**) (**B**) TEER (trans-endothelial electrical resistance), (**C**) Evans blue–albumin concentration, and (**D**) NaF were measured at normoxia and at 2, 4, and 6 h of hypoxia. Data are shown as mean ± SEM (*n* = 5–7). Quantitative analysis of (**B**) TEER, (**C**) Evans blue–albumin concentration, and (**D**) NaF in the brain endothelial cell monolayer was measured 4 h after hypoxia. Data are shown as mean ± SEM (*n* = 5–7). All experiments were pretreated with yuzu (1, 3, or 10 mg/mL) or HSP (3, 10, or 30 μg/mL) 30 min before hypoxia. * *p* < 0.01 vs. Nm, # *p* < 0.01 vs. Veh. Nm: normoxia; Veh: vehicle; HSP: hesperidin; Hx: hypoxia.

To investigate the effects of yuzu and HSP on BBB permeability during hypoxia in vitro, we measured BBB permeability in bEnd.3 cells using TEER, Evans blue, and NaF. We used Transwells to build an in vitro BBB model (Figure 2A). TEER values decreased significantly at 4 h after hypoxia (46.54 ± 1.56 Ωcm^2^) compared to normoxia (60.25 ± 1.98 Ωcm^2^). Treatment of bEnd.3 cells with yuzu (3 and 10 mg/mL) and HSP (10 and 30 μg/mL) prevented a hypoxia-induced decrease in TEER in a concentration-dependent manner; 55.42 ± 1.00 and 58.91 ± 0.91 Ωcm^2^ for yuzu, and 57.16 ± 0.94 and 59.22 ± 0.86 Ωcm^2^ for HSP, respectively (Figure 2B). The amounts of Evans blue and NaF in the lower chamber of the Transwell were greater in the hypoxic group (211.74 ± 8.34% and 132.92 ± 2.62%, respectively) than in the normoxia group (100%). The increases in Evans blue and NaF were maximally attenuated by treatment with 10 mg/mL of yuzu (137.24 ± 10.92% and 110.37 ± 5.09%, respectively) and 30 μg/mL of HSP (154.90 ± 13.01% and 103.79 ± 3.88%, respectively) (Figure 2C,D).

### 3.3. Effects of Yuzu and HSP on Disruption of Claudin-5 and ZO-1 during Hypoxia in bEnd.3 Cells

**Figure 3:**
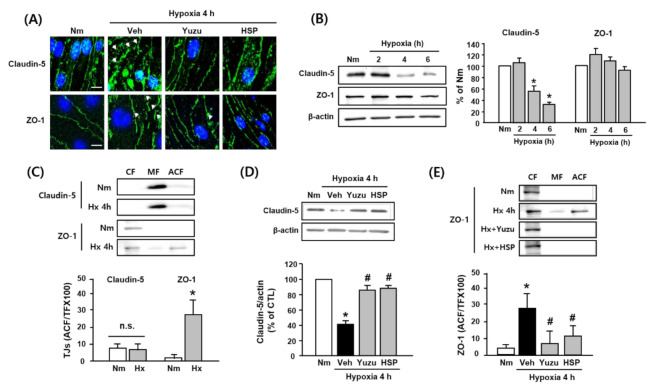
Effects of yuzu and HSP on disruption of claudin-5 and ZO-1 during hypoxia in bEnd.3 cells. (**A**) Representative fluorescence images of disruption of claudin-5 and ZO-1 induced by 4 h of hypoxia in brain endothelial cells. Arrows indicate regions of tight junction protein disruption. Green: claudin-5- or ZO-1-conjugated FITC, blue: Hoechst. Scale bar, 20 µm. (**B**–**E**) Western blotting of claudin-5 and ZO-1. (**B**) Protein levels of claudin-5 and ZO-1 during hypoxia (2, 4, and 6 h). (**C**) Redistribution of claudin-5 and ZO-1 during at 4 h of hypoxia in each fraction of bEnd.3 cells. (**D**) Effect of yuzu and HSP on degradation of claudin-5 at 4 h of hypoxia. (**E**) Effect of yuzu and HSP on redistribution of ZO-1 at 4 h of hypoxia. Data are shown as mean ± SEM. (*n* = 5–7) * *p* < 0.05 vs. Nm, # *p* < 0.05 vs. Veh. Nm: normoxia; Veh: vehicle; H: hypoxia; HSP: hesperidin; CF: cytosolic fraction; MF: membrane fraction; ACF: actin cytoskeleton fraction.

We investigated the effects of yuzu and HSP on the disruption of claudin-5 and ZO-1, major TJs in BBB, during hypoxia in bEnd.3 cells. As shown in Figure 3A, claudin-5 and ZO-1 were significantly disrupted after 4 h of hypoxia, which was prevented by pretreatment with yuzu (10 mg/mL) and HSP (30 μg/mL). To determine whether hypoxia affects the protein levels of claudin-5 and ZO-1, western blotting was performed. Consistent with our previous study, claudin-5 was significantly degraded after 4 h of hypoxia (52.14 ± 12.24%) as compared to the control, but ZO-1 was unchanged at the level of the protein (Figure 3B). On the other hand, the protein level of ZO-1 in the CF was significantly decreased and that in the ACF was increased after 4 h of hypoxia, indicating that translocation of ZO-1 from the CF to the ACF occurs (Figure 3C). Yuzu and HSP reduced both the degradation of claudin-5 and redistribution of ZO-1 during hypoxia (Figure 3D,E). These results suggest that the protective effects of yuzu and HSP against BBB dysfunction may be at least partially associated with the amelioration of the structural disruption of claudin-5 and ZO-1 during hypoxia.

### 3.4. Protective Effects of Yuzu and HSP on BBB are Associated with Antioxidant Activity in bEnd.3 Cells

**Figure 4:**
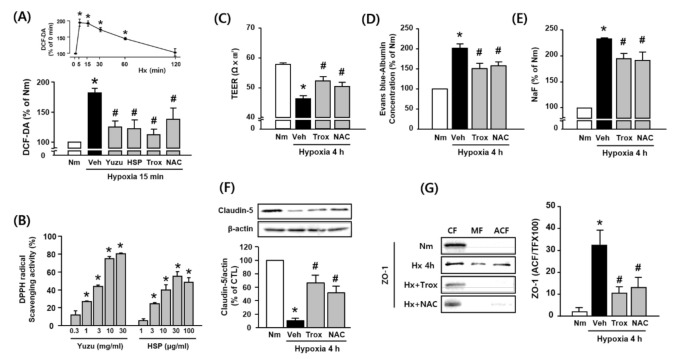
Protective effects of yuzu and HSP on the disruption of claudin-5 and ZO-1 are associated with antioxidant activity during hypoxia in bEnd.3 cells. (**A**) ROS generation during hypoxia was quantified by pretreating the cells with yuzu, HSP, Trolox, or NAC. ROS levels in the cells were quantified by measuring DCF-DA (2′7′-dichlorofluorescein diacetate) fluorescence intensity and are represented as the percentage (%) intensity at 0 min. (**B**) DPPH radical scavenging activity of yuzu and HSP in vitro. * *p* < 0.05 vs. negative control (methanol alone). (**C**–**E**) Quantitative analysis of (**C**) TEER, (**D**) Evans blue–albumin concentration, and (**E**) NaF in the bEnd.3 cell monolayer, as measured 4 h after hypoxia. All cells were pretreated with 100 μM Trolox and 100 μM NAC 30 min before hypoxia. (**F**,**G**) Western blotting of claudin-5 and ZO-1 in the absence or presence of Trolox or NAC during 4 h of hypoxia. Data are shown as mean ± SEM (*n* = 5–7). * *p* < 0.05 vs. Nm. # *p* < 0.01 vs. Veh. Nm, normoxia; Veh, vehicle; ROS: hydrogen peroxide; Hx, hypoxia; HSP, hesperidin; CF, cytosolic fraction; MF, membrane fraction; ACF, actin cytoskeleton fraction; Trox, Trolox.

As shown in Figure 4A, hypoxia increased the levels of ROS (hydrogen peroxide), and this effect was significantly inhibited by yuzu and HSP as well as by well-known antioxidants Trolox and NAC in bEnd.3 cells. In the absence of hypoxia, treatment with yuzu or HSP alone did not increase the level of ROS (data not shown). Moreover, yuzu and HSP showed a DPPH radical scavenging activity in a concentration-dependent manner (Figure 4B). The decrease in TEER and the increase in extravasation of Evans blue and NaF were attenuated by Trolox and NAC during hypoxia (Figure 4C–E). In addition, Trolox and NAC prevented the degradation of claudin-5 and redistribution of ZO-1 during hypoxia 4 h (Figure 4F,G). These results suggest that the protective effects of yuzu and HSP on BBB permeability and TJ disruption may be associated with their antioxidant activities.

### 3.5. Protective Effects of Yuzu and HSP on FoxO3a/MMP-Mediated Claudin-5 Degradation are Associated with their Antioxidant Activity in bEnd.3 Cells

**Figure 5:**
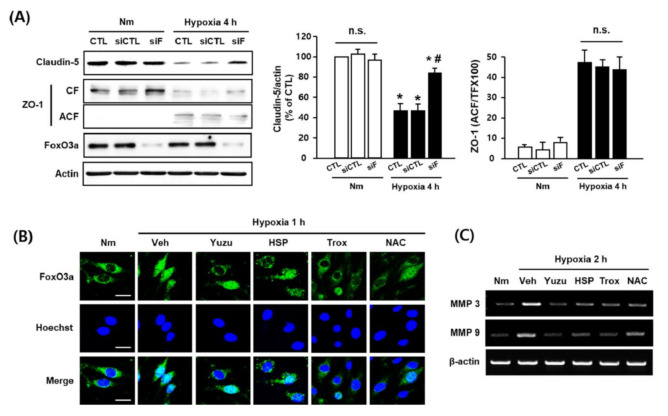
Protective effects of yuzu and HSP on FoxO3a/MMP-mediated claudin-5 degradation via antioxidant activity in bEnd.3 cells. (**A**) Effect of FoxO3a silencing on claudin-5 and ZO-1. Quantitative analysis of degradation of claudin-5 and redistribution of ZO-1 in bEnd.3 cells; bEnd.3 cells were transfected with siRNA control (siCTL) or siRNA FoxO3a (siF), as described in the Materials and Methods. The siCTL- or siF-transfected bEnd.3 cells were incubated for 4 h under normoxia or hypoxia. Samples were lysed and immunoblotted for claudin-5, ZO-1 (CF and ACF), FoxO3a, or actin; *n* = 5, data are shown as mean ± SEM, * *p* < 0.05 vs. Nm, # *p* < 0.05 vs. siCTL of hypoxia. (**B**) Immunofluorescent staining of FoxO3a (green) and Hoechst (blue) in bEnd.3 cells. Scale bar, 20 μM; *n* = 4. (**C**) Expression of MMP-3/9 mRNA levels in bEnd.3 cells. The mRNA levels of MMP-3/9 were measured at hypoxia, 2 h in the absence or presence of yuzu, HSP, Trolox, or NAC. Nm, normoxia; Veh, vehicle; Hx, hypoxia; HSP, hesperidin; CF, cytosolic fraction; ACF, actin cytoskeletal fraction; siCTL, siRNA control; siF, siRNA FoxO3a; Trox, Trolox.

In our previous study, we suggested that the translocation of FoxO3a induces degradation of claudin-5 via an increase in MMP-3/9 mRNA levels [26]. Based on our observations in this study, we investigated the effects of yuzu and HSP on the translocation of FoxO3a and an increase in MMP-3/9 mRNA levels as potential mechanisms of TJ disruption during hypoxia. We used siFoXO3a and observed that FoxO3a regulates claudin-5 degradation by inhibiting the process. However, we found no significant difference in ZO-1 redistribution between the control and siFoxO3a groups during hypoxia (Figure 5A). Fluorescence imaging revealed that the FoxO3a transcription factor moved into the nucleus in the hypoxia group, but not in the control group, and that this translocation was inhibited by yuzu (10 mg/mL), HSP (30 μg/mL), Trolox (100 μM), and NAC (100 μM) (Figure 5B). The mRNA levels of MMP-3/9 were greater in the hypoxia group than in the control group, and these increases were prevented by treatment with yuzu, HSP, trolox, or NAC (Figure 5C). These results indicate that the protective effects of yuzu and HSP on claudin-5 degradation during hypoxia occur via the inhibition of FoxO3a translocation and MMP3/9 activation, suggesting that this process is associated with their antioxidant activities in bEnd.3 cells.

## 4. Discussion

The present study demonstrates that yuzu and its major component HSP attenuate BBB dysfunction during hypoxia by inhibiting claudin-5 degradation and ZO-1 redistribution through antioxidant activity.

VCI is known to be a complex spectrum resulting not only from an acute event such as stroke but also from progressive small vascular injury and, therefore, therapeutic approaches for VCI should be different [6]. Although the extent to which vascular lesions affect VCI is not well known, the treatment of vascular lesions can pave the way for successful treatment of VCI, because cognitive impairment often occurs in patients with atherosclerosis, cerebral amyloid angiopathy, or cerebral hemorrhage [30,31]. In addition, VCI is reported to be attributed to cerebrovascular injury such as hypoxia/ischemia-induced BBB damage in the brain [32], suggesting the maintenance of BBB integrity as a potential therapeutic target for VCI. However, a lack of understanding of the mechanisms for VCI has thus far limited the development of therapeutics targeting BBB [9]. TJs are complexed between microvascular endothelial cells, and their function is particularly important for maintaining BBB integrity [33]; BBB integrity is compromised when TJs open. TJs are constructed by a complex network of proteins, such as occludin, claudin, junctional adhesion molecules (JAMs), and ZO-1, -2, and -3. Among them, claudin-5 and ZO-1 were found to be particularly important in maintaining junction assembly of BBB endothelial cells, and their absence correlates with BBB disruption [34,35,36]. In fact, knockdown of claudin-5 results in increased permeability of BBB endothelial cells [34]. Since ZO-1 serves as a support structure for signal transduction proteins and as a recognition protein for TJ placement [22], alteration of ZO-1 has been suggested to contribute to BBB permeability [37]. Furthermore, it has been reported that hypoxia-induced disruption of TJs can be caused not only by degradation but also the redistribution of TJs in BBB endothelial cells. Several studies have demonstrated that claudin-5 is degraded during hypoxia in mouse BBB endothelial cells, whereas others have shown that redistribution rather than degradation occurred during oxygen–glucose deprivation [38]. In addition, hypoxia-induced degradation of ZO-1 has been reported in rat BBB cells, whereas its redistribution has been shown in the mouse and pig BBB [39,40]. Consistent with previous reports, our results in the present study indicated that claudin-5 is degraded and ZO-1 is redistributed during hypoxia in b.End3 cells. However, our results also contradict some studies that reported redistribution of claudin-5 and degradation of ZO-1 during hypoxia. These controversial results may be due to different experimental conditions, such as hypoxic exposure times, and the cellular systems.

Oxidative stress is a known common feature of various pathological conditions that induce BBB disruption [41]. Indeed, many reports suggest that ROS are key mediators of BBB breakdown, implicating antioxidants as potential neuroprotectants against BBB-associated brain damage. ROS have also been suggested to provide a common trigger for many downstream pathways that directly mediate BBB dysfunction, such as TJ modification, and MMP activation [41,42]. In BBB endothelial cells, it has been shown that ROS causes DNA damage by induction of cell death, membrane lipid peroxidation, and increased permeability [43,44]. Consistent with these previous reports, our present study has shown that the increase in BBB permeability and the disruption of claudin-5 and ZO-1 are likely mediated by ROS in bEnd.3 cells. This observation further supports the concept that antioxidants can serve as potential protective and therapeutic agents in BBB-associated pathological conditions [41].

Several studies using various disease animal models have demonstrated that yuzu has antioxidant activities [16]. Indeed, it is well known that a major component of yuzu, HSP, prevents oxidative stress in both in vitro and in vivo hypoxia models [16]. In the present study, we observed that hypoxia-induced ROS increase was almost completely inhibited by yuzu and HSP, and TJ integrity was significantly preserved by yuzu and HSP during hypoxia, indicating that the protective effects of yuzu and HSP mark them as potential ROS scavenging agents against BBB dysfunction. We further evaluated the potency of yuzu and HSP by using EC_50_ (50% effective concentration) values for BBB permeability. As a result, HSP seems to be approximately 200~1000 times more potent than yuzu as per simple comparison (Table S2). Considering that the content of HSP in yuzu is about 0.1% (Table S1), the effect of yuzu on BBB permeability is likely to be largely attributed to that of HSP.

Next, we sought to elucidate the downstream mechanisms of ROS for the protective effects of yuzu and HSP against BBB dysfunction, focusing on the disruption of TJs. In our previous study, we demonstrated hypoxia-induced upregulation of MMP-3/9 as an upstream pathway for claudin-5 degradation in bEnd.3 cells [26]. These changes were shown to be associated with alterations in FoxO3a phosphorylation. FoxO3a, a member of the FoxO subfamily, has been implicated in the regulation of the expression of genes involved in apoptosis and oxidative stress; suppression of FoxO3a was reported to exert neuroprotective effects against ischemic injury in the brain [45,46,47,48,49]. It has also been reported that functional activation of MMP-9 via FoxO3a-mediated MMP-3 activation is involved in the regulation of endothelial cell survival and vascular integrity [50]. The results from both our previous study and others show that hypoxia-induced TJ disruption is significantly reversed in the presence of siFoxO3a, further supporting a causal relationship between FoxO3a and BBB integrity during hypoxia [26]. It is known that MMP activation is involved in the pathway of ROS signaling, wherein ROS interferes with BBB integrity by disrupting TJs [41]. In accordance with these results, our present study demonstrates for the first time a possible link between ROS and FoxO3a/MMPs in the regulation of claudin-5 integrity in BBB endothelial cells during hypoxia; we have shown that known antioxidants Trolox and NAC block FoxO3a/MMP-mediated degradation of claudin-5. Our findings suggest an important role for ROS in regulating BBB integrity during hypoxia as an upstream signaling pathway for FoxO3a-mediated degradation of claudin-5 and redistribution of ZO-1. Many natural compounds are known to be effective not only in neurodegenerative disorders, but also in VCI, through antioxidant mechanisms [51,52,53]. Consistent with our results, some of the natural flavonoids, such as epicatechin, cyanidin, and HSP, have been reported to have a neuroprotective effect through BBB protection, including permeability [53]. However, the protective effect of yuzu on BBB dysfunction has never been evaluated previously. Moreover, the mechanism for the protective effects of yuzu/HSP on BBB dysfunction in terms of ZO-1 translocation and FoxO3a/MMPs-mediated claudin-5 degradation has been performed for the first time in this study.

## 5. Conclusions

The findings highlighted here seem to be very encouraging, but there are some limitations: in this study, we identified only two out of the four peaks of HPLC for yuzu extract (one peak as HSP and another as naringin) because the other two peaks, including the highest, could not be identified in our system (Figure S1). HSP and naringin are both phenolic compounds, but the possible involvement of non-polyphenolic components in yuzu could be ruled out. Even though this study focused on HSP in view of functional study and analysis, further research on naringin and other unidentified compounds remains to be investigated. In conclusion, our results indicate that the protective effects of yuzu and HSP against BBB dysfunction are, at least partially, associated with their inhibitory effects on the degradation of claudin-5 and redistribution of ZO-1, and that these effects mainly manifest through their antioxidant mechanisms. Our findings may contribute to therapeutic strategies for BBB-associated neurodegenerative diseases, including VCI.

## Supplementary Materials

The following are available online at https://www.mdpi.com/2076-3921/9/9/843/s1, Figure S1: Chromotographic result for ethanolic yuzu extract with detection at 280 nm, Figure S2: The structure of HSP. Table S1: Contents of HSP in ethanolic extract of yuzu. Table S2: The values of EC_50_ of yuzu and HSP against BBB permeability. Supplementary Method.
