# Supplementary material for: Yuzu and Hesperidin Ameliorate Blood-Brain Barrier Disruption during Hypoxia via Antioxidant Activity
Source: Antioxidants (Basel). 2020 Sep 9;9(9):843. doi: 10.3390/antiox9090843 (PMC7555663; doi:10.3390/antiox9090843)
Supplement: Supplementary file 1 [file antioxidants-09-00843-s001.pdf]

antioxidants

Supplementary Figures & Table

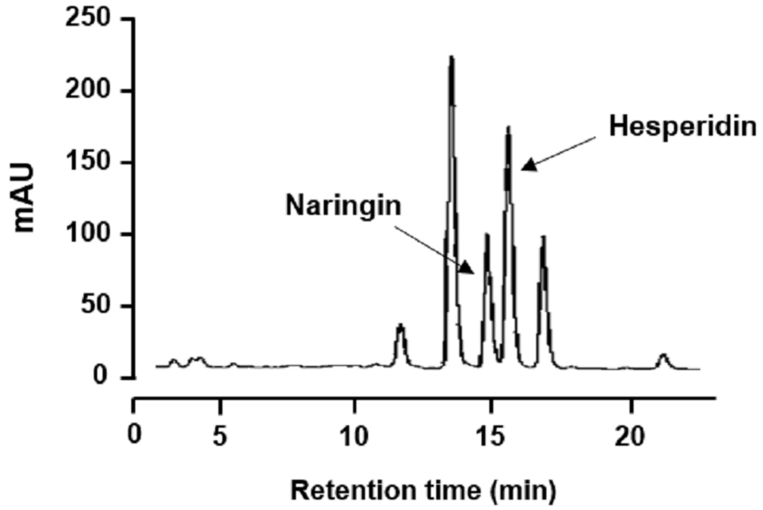

Figure S1. Chromotographic result for ethanolic Yuzu extract with detection at 280 nm.

Table S1. Contents of hesperidin (HSP) in ethanolic extract of Yuzu.

	Hesperidin	Naringin
Contents (mg/g of F.wt Yuzu)	0.97 ± 0.08	0.31 ± 0.03

All samples were tested in triplicate. Data are means ±SDs. F.wt, fresh weight

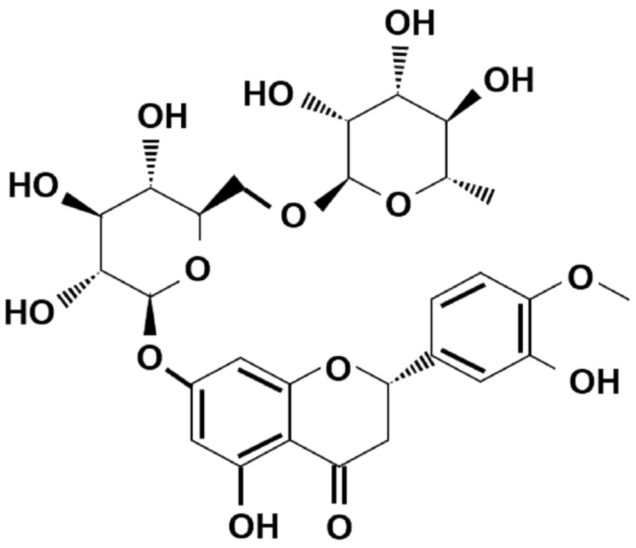

Figure S2. The structure of hesperidin.

Supplementary methods

Extraction of Yuzu and quantitative HPLC analysis

A mixture of 5 g (fresh weight) of sample and 25 mL of ethanol-DMSO (V/V; 50:10) was homogenized in a tube, and stirred on a magnetic stirrer for 10 min. Then 1 mL of the sample was centrifuged at 10,000g for 15 min at 4°C. The supernatant was collected and filtered through a 0.45 μm membrane filter before HPLC analysis. Reversed phase HPLC was used to assay hesperidin and naringin concentrations. An agilent 1100 Series System (Japan) consisting of a binary pump (DE43618438) and UV detector (DAD: G1315B) was used. The column was a Mightysil RP-C18 column (250 x 4.5; 2.5 i.d.,), and the mobile phase was composed of (A) 2% acetic acid (aqueous) and (B) 0.5% acetic acid (aqueous)-acetonirile (v/v; 50:50). Gradient elution was performed as follows: 0 min, 95:5; 10 min, 90:10; 40 min, 60:40; 55 min, 45:55; 60 min, 20:80 and 65 min, 0:100. The mobile phase was filtered under vacuum through a 0.45 μm membrane filter before use, and a flow rate of 1 mL/min was used. UV absorbance was measured at 280 nm. The column temperature was maintained at 25°C.
